# Identification and Characterization of the Interaction Site between cFLIP_L_ and Calmodulin

**DOI:** 10.1371/journal.pone.0141692

**Published:** 2015-11-03

**Authors:** Gabriel Gaidos, Alexandra E. Panaitiu, Bingqian Guo, Maria Pellegrini, Dale F. Mierke

**Affiliations:** Chemistry Department, Dartmouth College, Hanover, NH, United States of America; Hungarian Academy of Sciences, HUNGARY

## Abstract

Overexpression of the cellular FLICE-like inhibitory protein (cFLIP) has been reported in a number of tumor types. As an inactive procaspase-8 homologue, cFLIP is recruited to the intracellular assembly known as the Death Inducing Signaling Complex (DISC) where it inhibits apoptosis, leading to cancer cell proliferation. Here we characterize the molecular details of the interaction between cFLIP_L_ and calmodulin, a ubiquitous calcium sensing protein. By expressing the individual domains of cFLIP_L_, we demonstrate that the interaction with calmodulin is mediated by the N-terminal death effector domain (DED1) of cFLIP_L_. Additionally, we mapped the interaction to a specific region of the C-terminus of DED1, referred to as DED1 R4. By designing DED1/DED2 chimeric constructs in which the homologous R4 regions of the two domains were swapped, calmodulin binding properties were transferred to DED2 and removed from DED1. Furthermore, we show that the isolated DED1 R4 peptide binds to calmodulin and solve the structure of the peptide-protein complex using NMR and computational refinement. Finally, we demonstrate an interaction between cFLIP_L_ and calmodulin in cancer cell lysates. In summary, our data implicate calmodulin as a potential player in DISC-mediated apoptosis and provide evidence for a specific interaction with the DED1 of cFLIP_L_.

## Introduction

cFLIP (cellular FLICE-like inhibitory protein) is a key anti-apoptotic protein over-expressed in multiple types of tumor cells [1, 2]. At high cytosolic concentrations, cFLIP inhibits extracellular receptor-mediated (or extrinsic) apoptosis, which in tumor cells enables a mechanism for cell survival and uncontrolled proliferation [3]. Moreover, cancer cells displaying high levels of cFLIP expression also appear to become resistant to chemotherapeutic agents [3–9]. In conventional chemotherapy, one aim is to induce cell death in tumor cells, but in many cases these cells display resistance to receptor-mediated apoptosis and cFLIP appears to be involved in this phenomenon [10–14]. For these reasons, cFLIP represents an attractive target in cancer therapy.

cFLIP exerts its anti-apoptotic effect by disrupting efficient formation of the Death Inducing Signaling Complex (the DISC), a large intracellular protein assembly through which the extrinsic apoptotic signaling pathway is activated [1, 15]. Normal activation of DISC-mediated apoptosis is achieved through homotypic protein-protein interactions (PPIs) between members of the Death Domain super-family including Fas, FADD, and procaspase-8/10. cFLIP is considered to interfere with these interactions by competitively removing procaspase-8 from the DISC [1–3, 7, 15–18]. The ubiquitous calcium-sensing protein calmodulin has also been implicated as an antagonist of the extrinsic apoptotic pathway and it has been shown that calmodulin antagonists sensitize cancer cells to apoptosis [19–21]. Given these observations and the growing evidence of calmodulin interacting with various DISC components, the existence of a protein-protein interaction between cFLIP and calmodulin has also been postulated [20, 22–24].

The proliferation promoting properties of cFLIP and calmodulin have been functionally linked in a number of cancer types [8, 9, 20, 23]. Several observations of calmodulin antagonists either inhibiting metastasis or stimulating TRAIL-mediated apoptosis have been reported [8, 25]. The calmodulin antagonist W7 was shown to reduce growth of solid sarcoma 180, B-16 melanoma, and Ehrlich ascites carcinoma, and it inhibited metastasis of Lewis lung carcinoma [25]. A number of cases where calmodulin antagonists improved retention and cytotoxicity of chemotherapeutic agents in resistant P388 cells have been described [23]. Tamoxifen and trifluoperazine (both potent calmodulin antagonists) induced apoptosis exclusively in Fas-positive cholangiocarcinoma cells, suggesting a functional link between calmodulin and Fas signaling [26]. It has been proposed that calmodulin interacts with the Fas death receptor in a Ca^2+^-dependent manner in Jurkat cells and osteoclasts undergoing Fas or calmodulin antagonist-induced apoptosis [22, 24]. However, a clear mechanistic explanation never emerged from these studies. Hwang et al. found that out of the 180 enzyme inhibitors they tested, the calmodulin antagonist fluphenazine-N-2-chloroethane alone enhanced caspase-8 activity in human lung cancer H1299 cells and, furthermore, inhibited binding between calmodulin and cFLIP [8]. A different study showed that trifluoperazine also inhibited the interaction between these two proteins in cholangiocarcinoma cells [21]. More recently it was reported that cFLIP_S_, a shorter isoform of cFLIP, is strongly upregulated in surviving non-small cell lung carcinomas (NSCLC) in response to chemotherapy, promoting TRAIL resistance. This study also showed that inhibiting calmodulin in these surviving cells leads to down-regulation of cFLIP_S_, which correlated with resensitization to TRAIL treatment [9].

The molecular mechanism of the synergistic effect of calmodulin and cFLIP on apoptotic activity has not yet been explained. Here we present biophysical and structural evidence that cFLIP interacts directly with calmodulin in a Ca^2+^-dependent manner, and the interaction is mediated by an amphipathic segment on the C-terminus of the cFLIP death effector domain 1 (DED1). This finding furthers our understanding of DISC function, and opens an opportunity to develop selective cFLIP inhibitors for cancer therapy. Targeting cFLIP as opposed to calmodulin would present a number of advantages and would greatly enhance treatment specificity, as blocking calmodulin could potentially give rise to a number of deleterious effects within cells and tissues, given the large number of processes involving Ca^2+^/calmodulin signaling, including cell cycle regulation, signal transduction through second messengers, and cytoskeleton formation [27–29]. The growing body of evidence that calmodulin interacts with all DISC components participating in the cFLIP-mediated apoptotic inhibition, including Fas, FADD, and as we show here cFLIP itself, may reshape the extrinsic apoptosis model and will further understanding of cancer cell pathology and therapeutic avenues.

## Results

### cFLIP_L_ binds calmodulin through the death effector domain 1 (DED1)

**Fig 1:**
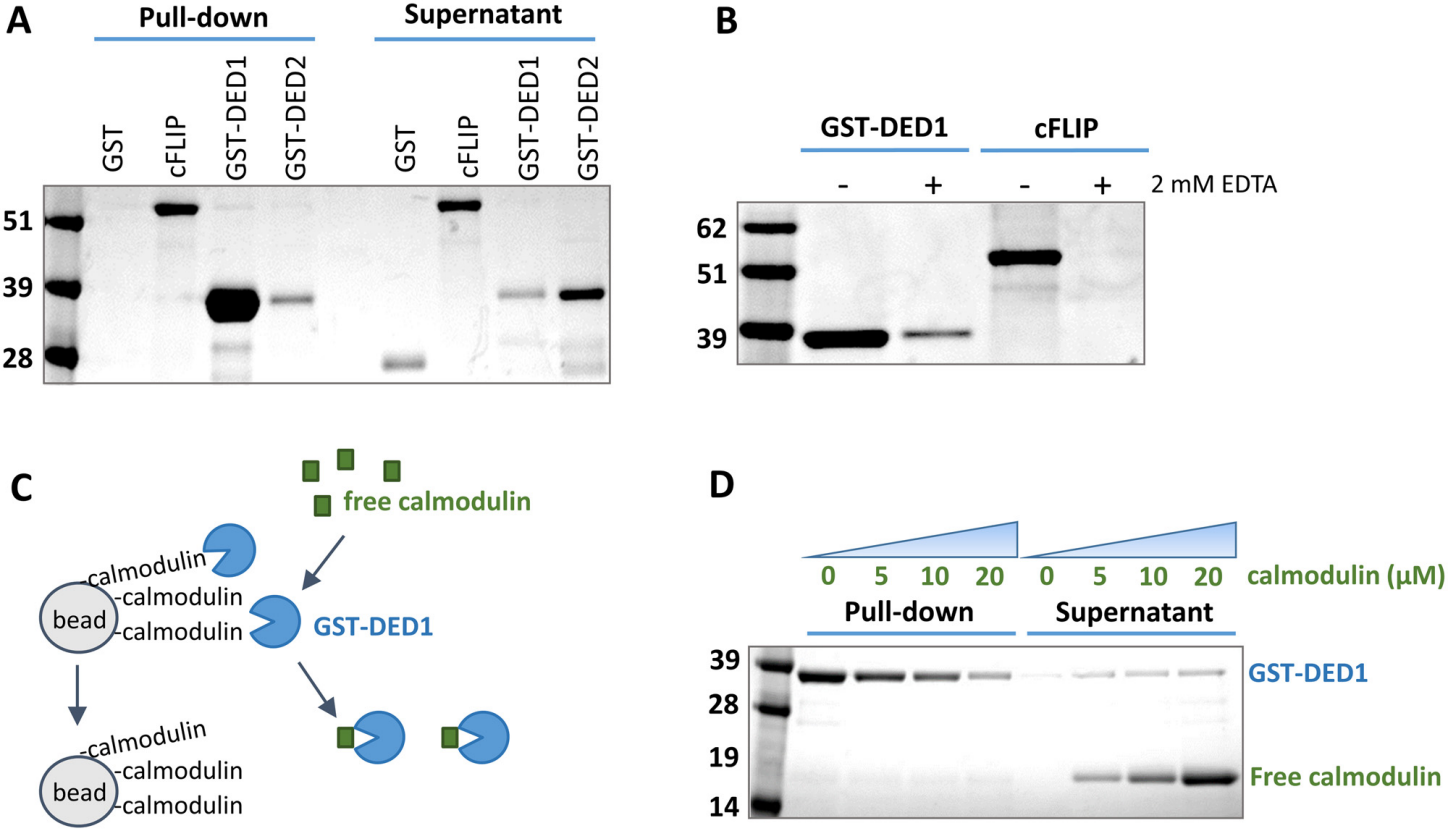
The cFLIP_L_/calmodulin interaction is mediated by the DED1 domain. Calmodulin conjugated on sepharose resin was used as bait in all pull-down assays and purified recombinant cFLIP_L_ or its DED domains were used as prey (10 g each). Pull-down lanes consisted of 50 μL resin and supernatant lanes of 500 μL buffer, rendering a dilution factor of 10 for non-interacting prey proteins in the SDS/Coomassie gel. **(A)** Full-length cFLIP_L_ pulls down on calmodulin-conjugated resin (lane 2), with DED1 strongly interacting with calmodulin (lane 3) while DED2 exhibits almost no interaction (lane 4). GST alone, used as a control, displayed no binding to calmodulin (lane 1). **(B)** The pull-down was repeated for DED1 and full-length cFLIP_L_ in the presence (lanes 1 and 3) or absence (lanes 2 and 4) of Ca^2+^. Neither cFLIP nor its DED1 interact with calmodulin in the absence of Ca^2+^ (lanes 2 and 4). Only pull-down lanes are shown. **(C)** To further confirm the specificity of the DED1/calmodulin interaction, a competition assay was carried out in the pull-down format, illustrated conceptually in this schematic. Recombinant GST-DED1 is incubated with calmodulin-conjugated resin in the presence of increasing amounts of free calmodulin, which is expected to compete with the resin for binding to DED1. **(D)** Results of assay described in **C**. Free calmodulin displaces DED1 from the resin in a dose-dependent manner.

Following reports of calmodulin acting as an antagonist of the TRAIL pathway, we queried the possibility of a specific interaction between calmodulin and the major DISC anti-apoptotic regulator, cFLIP. Full-length cFLIP_L_ and calmodulin were cloned and recombinantly expressed as polyhistidine-tagged proteins. The proteins were purified by affinity chromatography and characterized via size exclusion chromatography and SDS-PAGE. Protein-protein interactions were probed in a pull-down assay utilizing calmodulin-conjugated beads, which showed that full-length cFLIP_L_ interacts with calmodulin (Fig 1A, lane 2).

In order to identify the site on cFLIP_L_ that interacts with calmodulin, the two death effector domains (DEDs) were cloned, expressed, and purified separately as GST-tagged fusion proteins (S1 Fig). Both domains were tested for calmodulin binding in pull-down assays. As shown in Fig 1, DED1 is almost entirely captured on the calmodulin-conjugated resin (lane 3) while DED2 remains in the supernatant (lane 4). This suggests that the DED1 interaction with calmodulin is strong, while the interaction with DED2 is significantly weaker. Therefore, we focused our molecular mapping studies on DED1.

Since many calmodulin-binding events are regulated by Ca^2+^, we tested the effect of Ca^+2^ on the interaction between DED1 and calmodulin. We repeated the pull-down assay with both full-length cFLIP_L_ and GST-DED1 in the presence and absence of Ca^2+^. At a 2 mM concentration of the chelating agent EDTA, the cFLIP_L_/calmodulin interaction is almost completely inhibited, consistent with a calcium-dependent calmodulin binder (Fig 1B). To further confirm that DED1 is specifically interacting with calmodulin, we performed a *chase-off* competition assay in the pull-down format. Free recombinant calmodulin was titrated against a constant amount of DED1 bound to calmodulin-conjugated resin, in the presence of Ca^2+^ (Fig 1C). If DED1 is specifically binding to the calmodulin functionalization of the beads, free calmodulin should compete with the resin for binding and a dose response should be observed. As shown in Fig 1D, increasing amounts of free calmodulin gradually displace DED1 from the beads, clearly outlining a dose response, as hypothesized.

**Fig 2:**
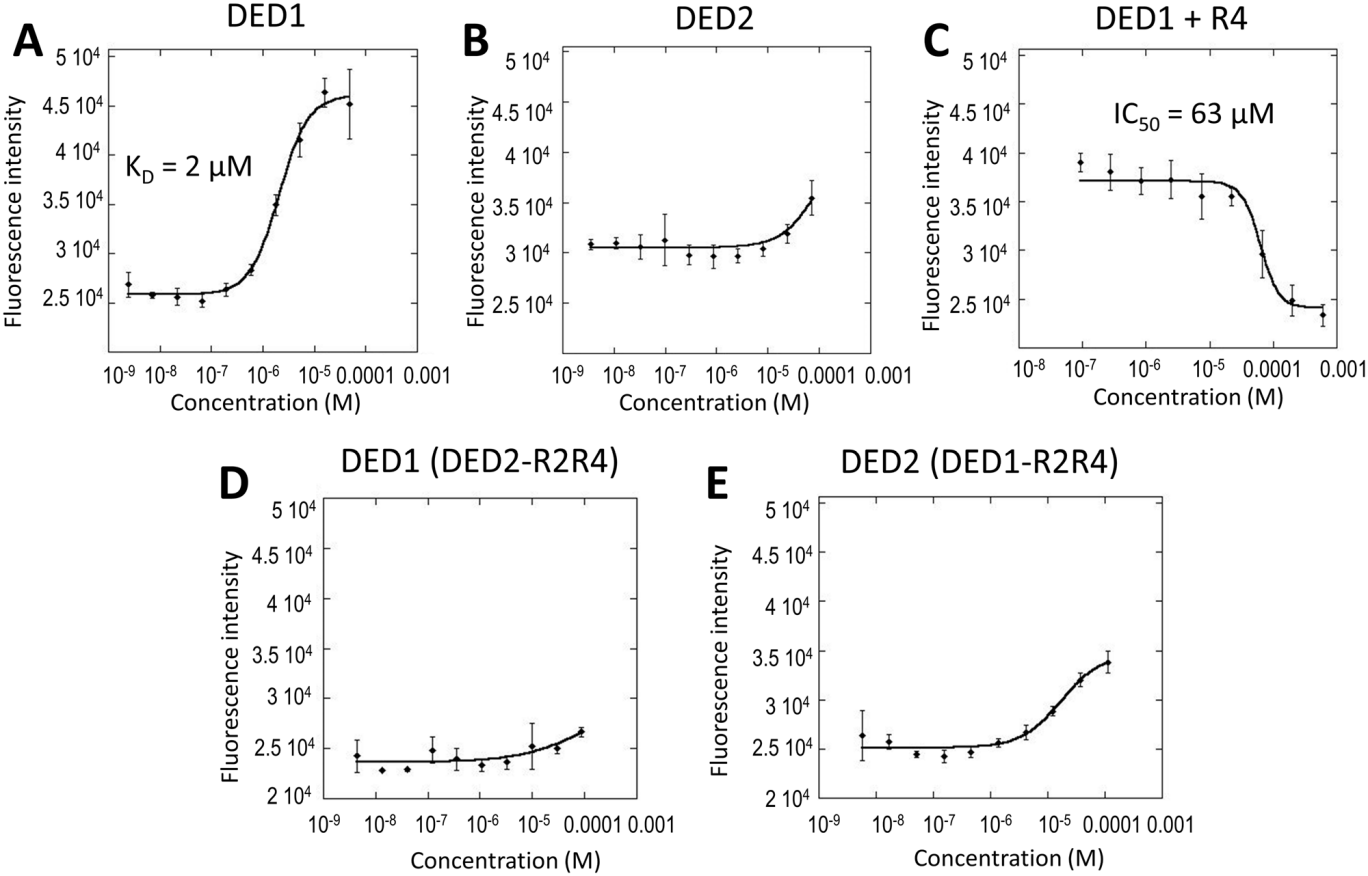
The interactions identified in pull-downs are recapitulated in ELISA experiments. In all ELISA experiments recombinant calmodulin was used as bait and immobilized, while GST-tagged DED1 or DED2 constructs were used as prey. **(A)** GST-DED1 was titrated and the affinity of the interaction with calmodulin was quantified. The two proteins interact with an affinity of 2 μM. **(B)** Binding of GST-DED2 is much weaker compared to DED1. **(C)** When GST-DED1 is kept at the K_D_ concentration, the R4 peptide competitively displaces DED1 from calmodulin. GST-DED1 was allowed to bind calmodulin, and then the R4 peptide was titrated. The R4 peptide binds with an IC_50_ of 63 μM. **(D) and (E)**: Swap of the R2R4 regions correlates with a loss of binding for DED1 and a gain of binding for DED2. DED1-(DED2-R2R4) exhibits diminished calmodulin binding, indicating a loss in activity (**D**) and DED2-(DED1-R2R4) displays a clear gain in calmodulin-binding activity (**E**). Error bars denote standard deviation of three replicates.

The DED1/calmodulin interaction observed in pull-down assays was also validated by ELISA. Calmodulin was immobilized on ELISA plates and GST-DED1 was added at increasing concentrations. Binding affinity was measured by monitoring the fluorescence intensity response of the anti-GST AlexaFluor 488 conjugated antibody. Curve fitting of fluorescence intensity as a function of analyte concentration yielded a K_D_ of 2 μM for the interaction between calmodulin and GST-DED1 (Fig 2A). By contrast, the GST-DED2 titration did not result in a binding curve within the concentration range utilized (Fig 2B).

### The calmodulin interaction site is located at the C-terminus of cFLIP DED1

The amino acid sequence of DED1 was BLAST screened against the Calmodulin Target Database in search of potential sequence homology with validated calmodulin binding peptides [30]. Fifty-nine homologous sequences clustered onto the C-terminal part of DED1, indicating the potential location of the binding site (S2A Fig). These DED1 segments were labeled as regions 1 through 4, of which regions 2 and 4 (R2, R4) were of most interest based on the large number of hits from the screen, and the conservation of hydrophobic and positively charged residues (S2B Fig). We defined region 2 (R2) as the sequence spanning R^38^-S^51^, region 3 (R3) as L^55^-D^66^, and region 4 (R4) as V^62^-K^73^. Regions 3 and 4 have an overlap of five residues, as a clean demarcation could not be deduced due to overlapping results from the BLAST search (S2A Fig).

**Fig 3:**
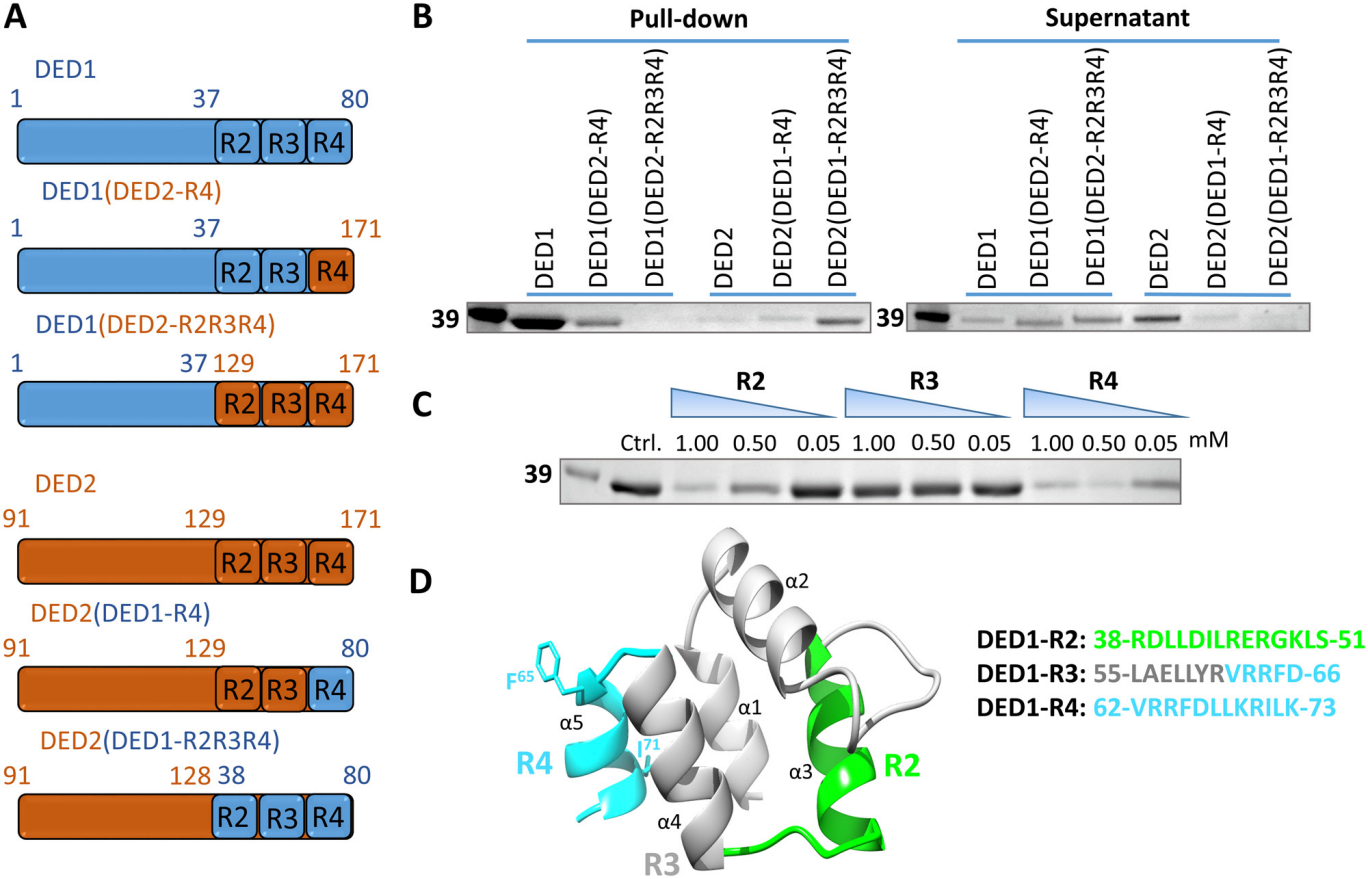
The interaction with calmodulin is mediated by the C-terminal part of cFLIP DED1. **(A)** Schematic representation of the C-terminal swaps between DED1 and DED2. DED1 segments are illustrated in blue and DED2 segments in red. **(B)** Constructs illustrated in **A** were used as prey in a pull-down assay on calmodulin-conjugated resin. DED1 constructs containing DED2 segments lose the ability to bind calmodulin (lanes 1–3), while DED2 constructs containing DED1 segments show a gain in binding ability for calmodulin (lanes 4–6). **(C)** Synthetic R2, R3, R4 peptides corresponding to the DED1 regions shown in **A** were titrated into the pull-down assay to displace GST-DED1 from calmodulin-conjugated resin; R2 and R4 show inhibitory activity, while R3 is ineffective. Only pull-down fractions are shown for simplicity. **(D)** Homology model of DED1 highlights the position of the R2, R3, and R4 regions. Amino acid sequences for the three peptides are shown next to the model.

Since to date there are no available structures for the DEDs of cFLIP_L_ or its mammalian isoforms, we generated a homology model for cFLIP DED1 based on the structure of the DED1 of viral FLIP MC159 (PDB ID: 2BBR.1.A) using the SWISS-MODEL server [31, 32]. Inspection of this model reveals that the three regions identified through BLAST screening map in large extent to three putative helices in the C-terminal portion of DED1. These regions are highlighted on the homology model in Fig 3D. Based on their amphipathic helical projections, we targeted regions 2 and 4 of DED1 and designed DED chimeric proteins in which these regions in DED1 and DED2 were swapped. The corresponding R2–R4 regions on DED2 were delineated based on sequence alignment with DED1. The chimeric constructs are illustrated schematically in Fig 3A. Folding and stability of the hybrid constructs were confirmed by circular dichroism (S3 Fig). All constructs exhibit the predicted alpha helical character (S3A and S3E Fig) and are thermodynamically stable, as indicated by the elevated melting points (S3B–S3D and S3F–S3H Fig).

The affinity of the DED1/DED2 chimeric constructs to calmodulin was tested in pull-down assays. As predicted, swapping the R4 regions of the two domains decreases the affinity of the chimeric DED1 for calmodulin, while swapping the R2–R4 regions completely abolishes binding (Fig 3B, lanes 1–3). More significantly, incorporating the same DED1 regions into DED2 partially rescues binding to calmodulin. Once again, swapping the R4 region alone weakly increases binding to calmodulin, while swapping the entire R2–R4 region for its DED1 counterpart greatly enhances binding of the chimeric DED2 to calmodulin (Fig 3B, lanes 4–6). This result was further validated by ELISA: the chimeric DED1-(DED2-R2R4) loses its ability to bind to calmodulin, while the chimeric DED2-(DED1-R2R4) gains moderate calmodulin-binding (Fig 2D and 2E).

### The C-terminal DED1 R4 peptide is a novel calmodulin binder

To further validate the cFLIP_L_/calmodulin binding epitope mapping, synthetic peptides corresponding to DED1 regions R2, R3, and R4 were tested in a competition pull-down assay for their ability to displace the calmodulin-bound DED1. The R4 peptide had the strongest inhibitory activity, followed by the R2 peptide. The R3 peptide had no visible effect on the interaction (Fig 3C). The R4 peptide acted as a competitive inhibitor of the DED1/calmodulin interaction in ELISA. Titration of the R4 peptide completely displaced DED1 from immobilized calmodulin with an IC_50_ of 63 μM (Fig 2C).

**Fig 4:**
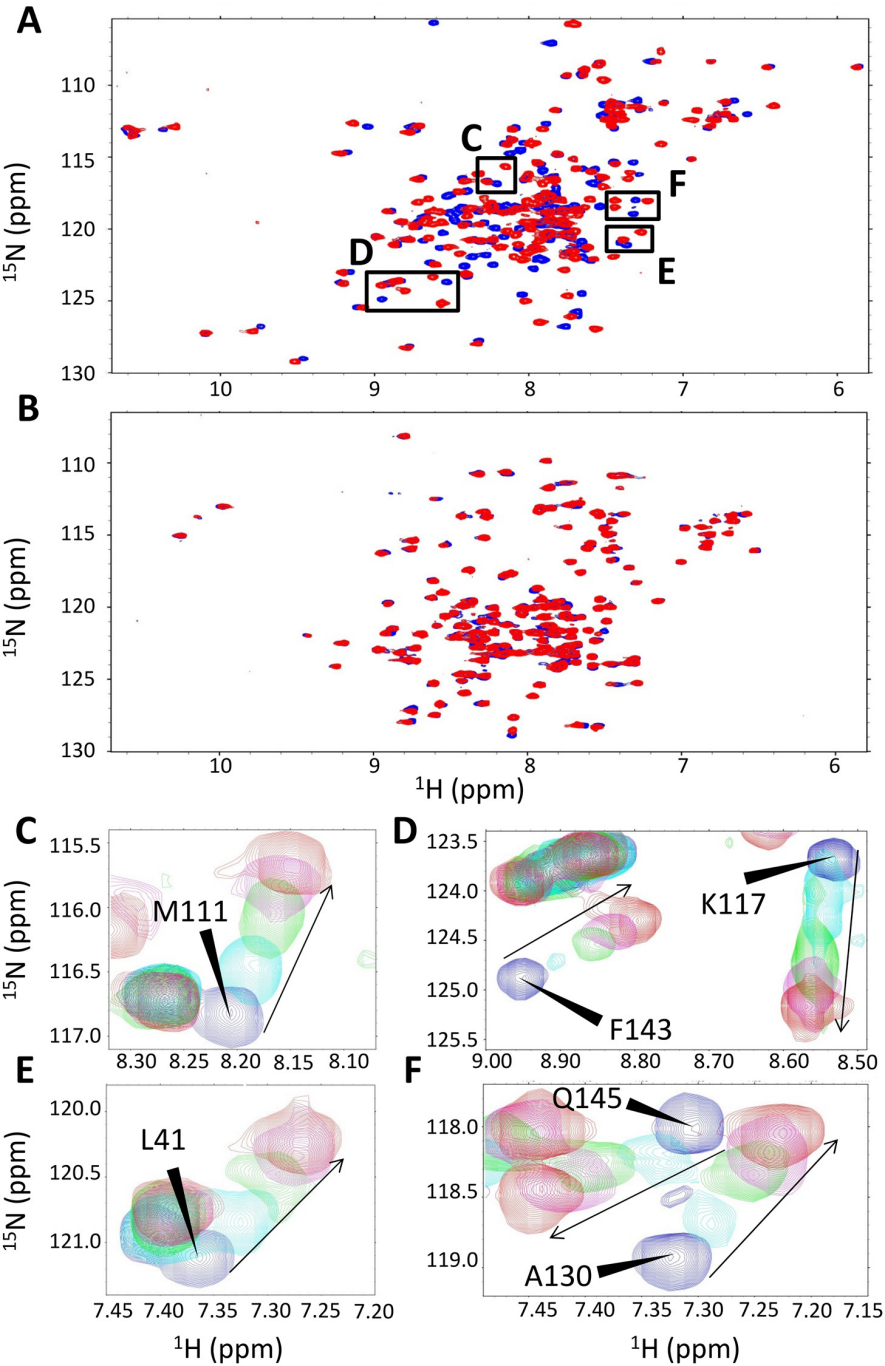
The DED1 R4 peptide interacts with calmodulin. **(A)** ^1^H, ^15^N-HSQC spectrum of calmodulin before (blue) and after addition of R4 (red), in the presence of Ca^2+^. The peak shifts between the two spectra are indicative of a binding event. **(B)** Chelation of Ca^2+^ with EDTA abolishes the interaction. The spectra shown in blue and red are in the absence or presence of excess R4, respectively. The high degree of spectral overlap indicates there is no interaction between R4 and the protein. **(C)-(F)** Enlarged views of corresponding insets from panel **A**, showing examples of residues directly involved in binding to R4. Spectra depict the following R4:calmodulin ratios: blue—0:1; cyan—1:1; green—1.5:1; magenta 2:1; red—5:1. Other titration points were excluded for ease of visualization. Arrows indicate the direction in which the peaks shift.

**Fig 5:**
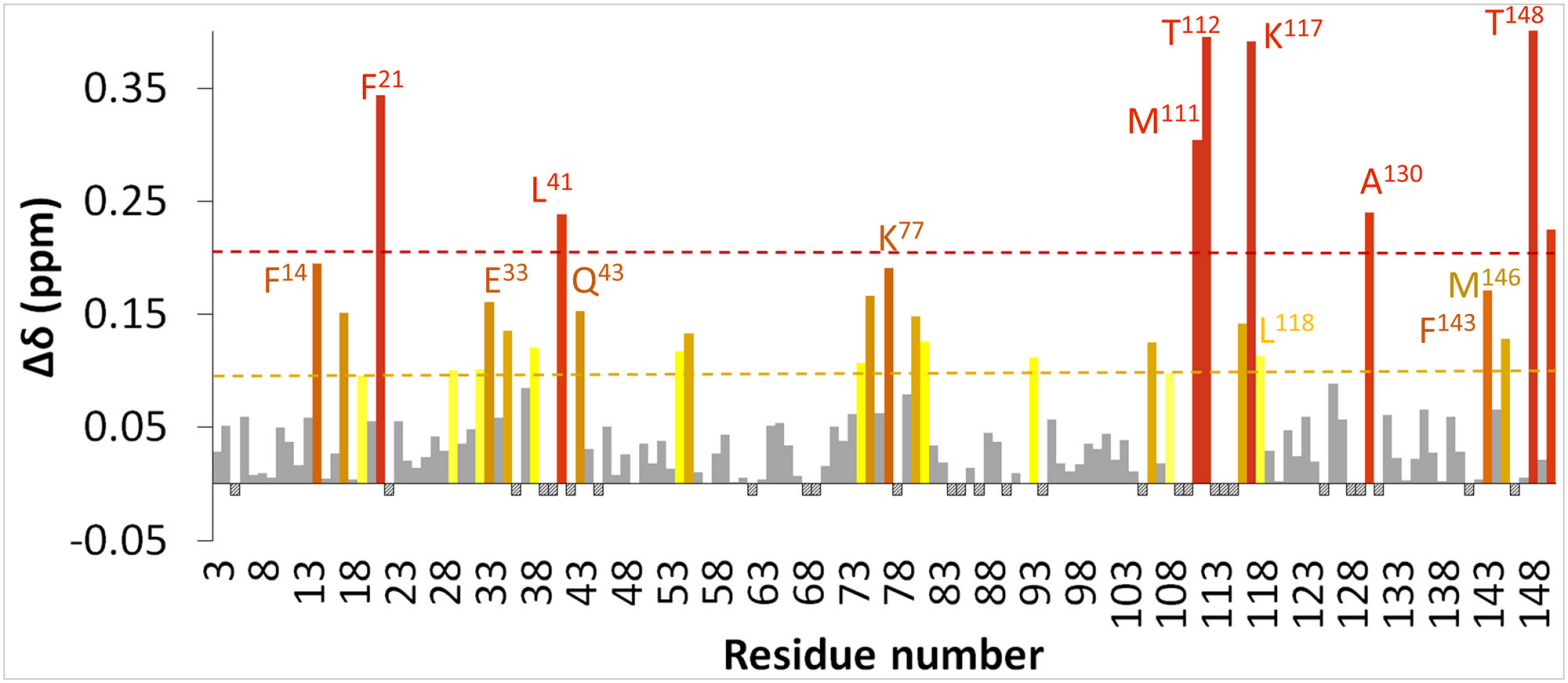
Calmodulin binding epitope for R4 peptide. Chemical shift perturbations (CSP) observed on the amino acid residues of ^15^N-labeled calmodulin upon R4 peptide binding. The magnitude of the CSP is plotted for every calmodulin residue. Eight residues experience shifts larger than two standard deviations (red). Eighteen additional residues experience shifts larger than one standard deviation (orange-yellow). Negative bars indicate residues that could not be unambiguously assigned.

To characterize the binding interface on the calmodulin side of the complex, we performed ^1^H,^15^N-HSQC NMR titration experiments using ^15^N-labeled calmodulin and unlabeled R4 peptide (Fig 4A). In this type of experiment, the ^1^H,^15^N resonances of calmodulin are monitored as unlabeled ligand (in this case R4) is titrated. An interaction between the ligand and the target protein induces changes in the chemical environment around the protein residues affected by binding, which translates into perturbations of the chemical shifts of the respective signals in the ^1^H,^15^N spectrum [33]. Addition of the unlabeled R4 peptide induced significant chemical shift perturbations (CSP) in the ^1^H,^15^N-HSQC spectrum of calmodulin, which is indicative of a binding event. The interaction was shown to be Ca^2+^-dependent, as addition of chelating agents disrupted binding: the spectra of calmodulin alone and in the presence of saturating amounts of R4 are essentially identical in the presence of EDTA (Fig 4B). A six-point titration of R4 (ranging from 0:1 up to 5:1 ratios of R4:calmodulin) (Fig 4C-4F) demonstrated that the binding can be saturated and yielded a K_D_ value of 58–62 M, in agreement with the IC_50_ determined from the ELISA competition experiment. To determine the binding site for R4 on calmodulin, we obtained a nearly complete backbone assignment of calmodulin resonances (all non-Pro residues except Gly^1^, Met^2^, His^109^) using standard triple resonance experiments on a ^1^H,^13^C,^15^N-labeled sample of calmodulin (BMRB accession code 19630). The NMR resonance assignments available in the BMRB did not match either the protein sequence or the experimental conditions we utilized. Fig 5 displays a bar graph of CSPs mapped for each calmodulin residue in the presence of saturating R4 amounts. The calmodulin residues that experience statistically significat shifts upon binding of the R4 peptide include F^14^, F^21^, M^38^, L^41^, and E^33^ on the N-terminal lobe. Similarly, residues most affected by binding on the C-terminal lobe include M^111^, T^112^, E^116^, K^117^, L^118^, A^130^, F^143^, M^146^, T^148^, and K^150^. Other residues on the C-terminal lobe are clearly affected by binding, because the signals in the ^1^H,^15^N-HSQC spectrum broaden and shift to the extent where their assignment cannot be unambiguously transferred. These include L^114^, E^125^, E^129^, and E^141^. There are two main classes of calmodulin residues that are consistently described as participating in binding events with target peptides. These are either hydrophobic residues such as Phe, Leu, or Met in so-called FLMM cavities in both the N- and C-terminal calmodulin lobes (where the letters stand for the comprised amino acids) or charged Glu residues surrounding the FLMM cavities [34]. The FLMM residues anchor the ligand through conserved hydrophobic interactions and the glutamic acid sidechains stabilize the interaction through electrostatic contacts [35, 36]. These conserved interaction sites were first described extensively for calmodulin in complex with peptides M13, smMLCK, and CaMKII, and have since been reported for a large number of the three hundred calmodulin binders [30, 37–39]. Indeed, our CSP data are consistent with this canonical “*wrap-around*” calmodulin binding modality, where FLMM and charged Glu residues on both lobes participate in binding to R4, with a larger contribution from the C-lobe.

### Structure of R4 peptide bound to calmodulin

**Fig 6:**
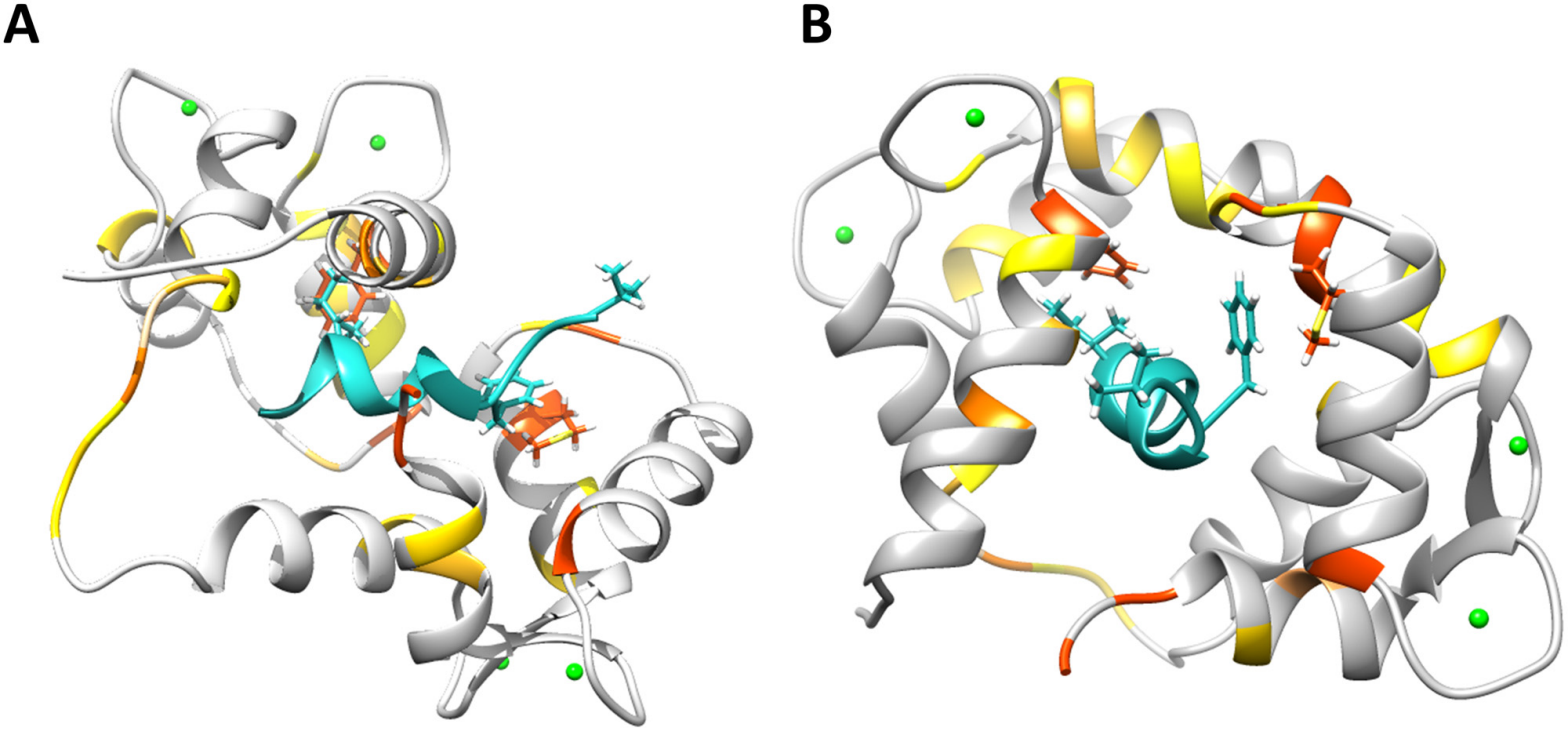
Model of R4 peptide/calmodulin complex. The solution structure of R4 was docked into calmodulin and the model was refined by molecular dynamics. Colored on calmodulin are the residues which undergo significant chemical shifts in the NMR titration experiments, ranging from red (strongest) to light yellow (weakest). The color scheme corresponds to that in Fig 5. The R4 hydrophobic anchors F^65^ and I^71^ are shown, as well as the calmodulin residues that experience the strongest CSPs, M^111^ and F^21^. **(A)** The model is oriented to facilitate visualization of the peptide in the pocket (the N-lobe of calmodulin is facing upward and the C-lobe downward). **(B)** 90° rotation of the model in **A**.

The NMR titration experiments described above demonstrated that the R4 peptide interacts with calmodulin with an affinity comparable to the value obtained from ELISA (ca. 50–60 μM). This affinity range typically correlates with a ligand off-rate fast enough to enable observation of transferred NOEs for the peptide in the presence of its binding partner [40, 41]. More precisely, in the unbound state small peptides are typically characterized by short correlation times and will exhibit very small, positive NOEs, or no NOEs. Upon binding to protein partners, NOEs generated in the bound state of the peptide are measured and analyzed in the unbound state [40, 41]. We therefore utilized transferred NOESY experiments to determine the structure of the R4 peptide bound to calmodulin. An ^15^N-labeled calmodulin sample was used in combination with ^15^N-filtered transferred NOESY pulse sequences, which allowed selective elimination of any protein-generated NOEs, thus enabling exclusive observation of peptide intra-molecular NOEs [42]. Sequential resonance assignment of the R4 peptide was achieved from additional TOCSY and ROESY experiments carried out for the peptide alone [43, 44]. From the transferred NOESY experiments we obtained 80 distance constraints (S2 Table), which were utilized in distance geometry calculations to produce the three-dimensional structure of R4 (PDB ID: 2N5R, BMRB ID: 25726). 97 structures were generated and the 21 structures corresponding to the lowest total energy were chosen for analysis. NOE violations and structure validation parameters are reported in S2 Table. A representative R4 peptide structure is shown in Fig 6, modeled in the binding pocket of calmodulin. The peptide itself is unstructured when not bound to calmodulin, as evidenced by the absence of any backbone amide ^1^H-^1^H NOE or ROE signals in the NOESY or ROESY spectra acquired for the peptide alone. However, upon binding calmodulin, the peptide adopts a mostly helical conformation through residues F^65^-L^72^ (Fig 6A). The N-terminal V^62^-R^64^ residues remain unstructured, as indicated by the absence of NOE signals in this region even in the bound state of the peptide.

The peptide solution structure was modeled in the binding pocket of calmodulin (PDB ID: 1QTX, calmodulin bound to the sMLCK smooth muscle myosin light chain kinase peptide) and the complex was further refined using molecular dynamics (Fig 6). This calmodulin structure was chosen for modeling because it is representative of the 1–14 class of calmodulin binders and the R4 peptide seems to best fit in the basic 1-8-14 subclass. The R4 structure was initially manually docked to the calmodulin structure by superposition to the sMLCK peptide, aligning the hydrophobic anchors, and by consideration of calmodulin CSPs in the NMR titration experiments. In the resulting model (Fig 6), R4 is aligned with the N- and C-termini of calmodulin in an anti-parallel fashion, with F^65^ and I^71^ positioned in the conserved protein hydrophobic pockets. Existing structures of other calmodulin complexes where the binding peptides display higher affinity for the C-lobe than the N-lobe like R4 does exhibit anti-parallel alignment of the peptide with respect to calmodulin [36]. In our model, the R4 hydrophobic anchors make contacts with calmodulin residues M^111^ and F^21^, respectively. These two protein residues exhibited the strongest CSPs in the NMR titration experiment (Fig 5). Other protein residues that were strongly affected upon peptide binding experimentally, such as T^112^ or K^117^ (and all the ones between them whose assignment could not be unambiguously transferred due to severe broadening or overlap), appear in close proximity to the peptide F^65^ in our model. As described earlier, other protein amino acids affected by binding include hydrophobic FLMM residues and charged glutamic acid sidechains predominantly on the C-lobe of calmodulin. In our model, these residues appear in close proximity to the R4 peptide F^65^ anchor, which is consistent with the large CSPs observed experimentally for these residues. The peptide N-terminus is positioned slightly outside of the protein binding pocket and is likely unstructured, as also indicated by the absence of NOEs for this region. Fig 5 indicates that there is a second set of strong CSPs experienced by residues at the N-terminal lobe of calmodulin. In this model, these residues form the pocket which accommodates the second peptide hydrophobic anchor, I^71^. Additionally, the residues in the flexible linker adjoining the two calmodulin lobes also experience significant CSPs in the experimental data, even though they do not appear to make close contacts with R4 in our model. This linker is known to adopt a wide range of conformations to accommodate the different calmodulin binders [36, 37, 39]. Therefore, we suspect that in solution this linker either makes more intimate contacts with the C-terminus of R4 (L^72^ and K^73^) or experiences a conformational modification as a result of the change in angle between the two calmodulin lobes upon peptide binding, generating the CSPs observed in the experimental titration.

### The cFLIP/calmodulin interaction is recapitulated in cancer cell lysates

After characterizing the calmodulin/cFLIP DED1 interaction using purified recombinant proteins, we confirmed the interaction is maintained in the more physiologically-relevant environment of the cancer cell lysates. We used lysates of NCI-H2030 non-small lung cancer cells for pull-downs with calmodulin-conjugated beads. NCI-H2030 cells express the CFLAR gene, which encodes for cFLIP, but endogenous levels were too low for Western blot detection. As such, the lysates were enriched with recombinant cFLIP_L_ to ascertain whether the interaction with calmodulin would still be maintained in the presence of other cytoplasmic components, which could compete with either one or both proteins in the native cell environment. The calmodulin-conjugated beads pulled down full-length cFLIP_L_ and this interaction was found to be inhibited but not fully blocked by 1 mM EDTA. We also observed that 1 mM R4 peptide is able to partially inhibit the interaction between cFLIP and calmodulin in the cell lysate environment, although to a lesser extent than with purified components (S4 Fig vs. Fig 3C).

## Discussion

Here we report the molecular details of the interaction between cFLIP_L_ and calmodulin. Our results show that full-length cFLIP_L_ interacts with calmodulin in pull-down assays in a Ca^2+^-dependent manner, both *in vitro* using isolated purified proteins as well as in the more physiologically-relevant environment of lung cancer lysates. By sub-cloning and characterizing the individual cFLIP_L_ domains we have further shown that cFLIP_L_ binds calmodulin through its DED1 and have validated this finding through a suite of biochemical and biophysical assays, including pull-downs, ELISA, and NMR. Searching against the Calmodulin Target Database allowed for a narrowing down of the interaction site to two regions on DED1 (R2 and R4). Of the two, R4 is the center of the calmodulin interacting site, both within the context of the intact protein (as demonstrated by the DED1/DED2 chimeric constructs) and as an isolated peptide.

Most calmodulin binding peptides are classified into families based on shared motifs (e.g., 1–10, 1–14, 1–18 motifs), where key bulky hydrophobic residues are located at defined distances [19, 30, 45, 46]. The R4 peptide does not fit unequivocally into any putative family: the closest match is the basic 1-8-14 subclass, with the caveat that the R4 peptide is only twelve amino acids long. However, it does include basic N-terminal residues (R^63^R^64^) followed by bulky hydrophobic residues one and eight amino acid positions downstream (F^65^ and L^72^, respectively). It should also be noted that numerous “non-canonical” calmodulin binding peptides have been identified which do not match a particular motif, but share the common characteristics of amphipathic alpha helices with clusters of basic residues [30]. The boundaries of the R4 peptide were chosen empirically, therefore more optimized delineations are possible. For example, the 58-LLYRVRRFDLLKRILK-73 cFLIP sequence could be a closer match for the 1-5-8-14 calmodulin binding motif. However, the fact that 62-VRRFD-66 is a region of overlap between R4 and R3, which showed no binding towards calmodulin or any inhibitory effect on the interaction between the former and DED1 would seem to suggest that if the R4 peptide were to be elongated, it should be at its C-terminus.

This conclusion is also supported by the large number of calmodulin binding peptides showing homology to the C-terminal part of peptide R4 and by our structural NMR data for R4 bound to calmodulin. In the model of the R4/calmodulin complex the N-terminus of R4 is placed slightly outside the calmodulin binding pocket, and this region appears unstructured both in the model and in the NMR experiments. In order for calmodulin to bind to the isolated DED1 as well as to full-length cFLIP_L_, this C-terminal region of DED1 would likely undergo a conformational rearrangement so as to accommodate calmodulin. Such a conformational change could enhance accessibility to the F^65^ and I^71^ hydrophobic anchors (which are solvent-exposed in our homology model of cFLIP DED1) and could have long-range effects, for instance on R2. Since R3 does not show any inhibitory activity on the DED1/calmodulin interaction in any of our assays, we speculate that R3 serves as a “hinge region” around which DED1 rearranges to accommodate binding to calmodulin through the C-terminal R4 region. Such an induced fit upon binding is consistent with our observation that the R2 region enhances the affinity of DED1 for calmodulin. Our results differ from previous reports of the 197–213 segment of cFLIP_L_ as the interaction site for calmodulin [21]. Since segment 197–213 represents the linker region between DED2 and the caspase-like domain, its elimination could induce major allosteric effects in cFLIP_L_. This discrepancy may also be due to interference with other DISC components not identified in the cell lysate-based assays previously conducted [21].

cFLIP upregulation has been correlated with metastasis in numerous types of tumors [9, 47]. One such example is represented by non-small cell lung carcinomas, which are the leading cause of death among all cancer patients. For this reason, we chose to further study the cFLIP/calmodulin interaction in NCI-H2030 cancer cell lysates. The interaction between cFLIP_L_ and calmodulin is clearly detectable in these lysates, further adding to the growing body of literature postulating the potential involvement of calmodulin signaling in the DISC. Competition with cFLIP-derived peptides was not as effective as in the assays with purified components. The drop in inhibitory activity could be attributed to two factors: i) the inhibitory peptides can interact with other cell components, thus diluting the effective concentration available for cFLIP_L_ inhibition; ii) cFLIP can be recruited into the DISC via multiple interactions, such as with procaspase-8 and/or DR5, FADD, and thus targeting one contact point for inhibition will only partially inhibit cFLIP recruitment.

Recently Majkut et al. have reported a computational model for the mode of interaction between components in the tripartite complex comprising cFLIP, FADD, and procaspase-8, proposing that the 2 helix of cFLIP DED2 interacts with FADD in a groove formed by the 1 and 4 helices of its own DED [16]. A previous report proposed that FADD itself also interacts with calmodulin through helices 8–9 and 10–11 of its death domain (DD) [48]. Our observation that calmodulin interacts with cFLIP via a putative helix 5 in its DED1 is compatible with these previously reported interactions. Given the number of literature reports of calmodulin antagonists sensitizing Fas-mediated apoptosis in a number of tumor cells, as well as the growing number of reports demonstrating direct interactions between calmodulin and almost each individual DISC component (Fas, FADD, cFLIP), it is plausible to envision calmodulin playing a role in DISC formation and function [8, 20–24, 26, 45, 48, 49]. As more interactions between calmodulin and individual DISC members are demonstrated, precisely determining the role calmodulin plays in DISC signaling becomes an increasingly complex task.

Interestingly, the F^65^ hydrophobic anchor in the R4 peptide is at the center of DED1’s RxDL “charge triad”. These charge triad motifs are highly conserved in all DEDs and have been implicated in the homo- and heterotypic interactions between DED-containing proteins [1, 16, 50, 51]. Studies conducted on the viral FLIP MC159 suggested this triad from helix 6 of its own N-terminal DED is important for the protein’s anti-apoptotic activity [1]. We can therefore begin to speculate as to the role calmodulin might play in DISC formation and signaling—if the same charge triad in cFLIP DED1 plays a similar role in the inhibition of apoptosis as MC159, then calmodulin binding at this site could act to regulate apoptosis.

Further studies will be required to precisely characterize the sequence and regulation of each calmodulin binding step, but all these interactions support the idea that calmodulin could act to shift the equilibrium towards increased recruitment of cFLIP to the DISC, consequently inhibiting Fas-induced apoptosis. This model provides a starting point for a mechanistic explanation as to why calmodulin antagonists can promote Fas-mediated apoptosis. [19, 20] Furthermore, this model also raises the possibility of a new avenue for pharmacological targeting in tumor cells. Since some calmodulin antagonists have a stimulating effect on Fas-mediated apoptotic activity, inhibiting the calmodulin/cFLIP interaction by targeting cFLIP DED1 could aid in restoring DISC-mediated programmed cell death while at the same time avoiding the wide range of potentially deleterious side effects that could result from global calmodulin inhibition. Blocking the cFLIP/calmodulin interaction could also be more effective than targeting the interactions between calmodulin and other DISC components (such as Fas, for instance), especially in the case of tumor cells that become resistant to Fas-stimulation in response to chemotherapy, a mechanism which has been linked with increased recruitment of cFLIP to the DISC [9, 22]. The inhibition of the cFLIP/calmodulin interaction is an attractive target for the development of novel cancer therapeutics. The structural characterization of the complex of calmodulin with the cFLIP DED1 R4 peptide, as well as the structure determination of the cFLIP death effector domain 1 will pave the way for the rational design of targeted small molecule inhibitors of the cFLIP/calmodulin interaction.

## Materials and Methods

### Synthetic Peptides

(Tufts University Core Facility, Boston, MA) were solubilized in PBS and stored as 100 μM aliquot stocks at -20°C until used. Peptide concentrations were determined by weight, assuming 98% purity in the HPLC fractions.

### Construct design

The cFLIP_L_ template (canonical isoform 4, NCBI Reference Sequence: NP_001189445.1, courtesy of Dr. Roya Khosravi-Far, Harvard University) was amplified by PCR using primers which introduced an NdeI site at both ends. The NdeI sites represent the junction points to the pET21b+ vector, in which cFLIP_L_ was introduced upstream of the C-terminal polyhistidine tag. The forward aligned clones were selected by DNA sequencing. All cFLIP subdomains were cloned into the GST-expressing pGEX-6P-1 plasmid. Human calmodulin cDNA was purchased from OriGene (CALM2 gene, cat # SC125707) and similarly amplified by PCR to be introduced into a pET16b plasmid. In contrast to full-length cFLIP_L_, however, calmodulin and the cFLIP subdomain constructs were cloned using Agilent’s QuikChange II XL site-directed mutagenesis kit by using a PCR insertion method which precludes restriction enzymes and ligases. Briefly, the DNA primers (Integrated DNA Technologies, Coralville, IA) were designed to include a distal segment overlapping with the target plasmids and a proximal segment overlapping with the protein construct (See S1 Table for a list of plasmids and primers). In the first step, the proximal segments were used as primers to PCR-amplify DNA segments of target clones with overhangs corresponding to the plasmids. In the second step, the purified PCR products were used for site-directed mutagenesis-based insertion, where the inserts’ distal/overhanging segments defined the insertion points on the target plasmids. The cloned constructs were confirmed by DNA sequencing (Dartmouth College Molecular Biology Core Facility). Amino acid boundaries of the constructs are depicted in S1 Fig. Primers used for all cloned constructs are listed in S1 Table. Note that both the pET16b and the pET21b+ vectors were modified as follows: the pET16b vector was engineered to include a TEV (Tobacco Etch Virus protease) cleavage sequence between the N-terminal polyhistidine tag and the construct of interest; the pET21b+ vector was engineered to knock out the T7 tag (ASMTGGQQMG) and we refer to this vector as pET21b+ΔT7.

### Protein purification

All employed proteins were purified via standard FPLC techniques. The *E. coli* cultures were grown to an OD_600_ = 2 in Terrific Broth, and then induced with 0.2 mM IPTG for 16 hours at 20°C, reaching a final OD_600_ = 15–20. Cells were harvested by centrifugation at 4,000–5,000 rpm for 20–30 minutes and the pellets were resuspended in 30 mL FPLC binding buffer supplemented with a protease inhibitor cocktail tablet (Roche Complete) and a DNAse (Benzonase), using 2 mM MgCl_2_ as a cofactor. Cells were lysed using a French Pressure Cell and soluble protein fractions were cleared by centrifugation at 40,000–60,000 rpm. All constructs except full-length cFLIP were soluble. cFLIP was insoluble, so it was resolubilized from the pellet under denaturing conditions (8 M Urea) after lysis and subsequently refolded. The proteins from the lysed cultures were purified by means of affinity chromatography on GE Healthcare Akta Xpress FPLC systems. Briefly, cleared cell lysates containing the 6His-tagged or GST-tagged over-expressed proteins were separated on Ni^2+^-conjugated or glutathione-conjugated resins (GE Healthcare HisTrap HP and GSTrap 4B 5 mL columns), respectively. Size exclusion chromatography (SEC) on Superdex-200 or Superdex-75 HiLoad prep-grade columns (GE Healthcare) was used for removal of glutathione or imidazole, and for separating the monomeric or dimeric proteins from higher molecular weight species. The protein samples in PBS, pH 7.4, 2 mM DTT (the SEC running buffer) were stored as aliquots at -80°C until used.

^15^N-only or ^15^N,^13^C-labeled calmodulin was produced by gently harvesting 300 or 100 mL cell cultures, respectively (2,000 rpm for 10–15 minutes) once an OD_600_ = 3 was reached, followed by resuspnesion of the pellets in the same volumes of M9 medium containing ^15^NH_4_Cl (3 g/L), and ^13^C-D-glucose (10 g/L) for the double-labeled sample. After resuspension, an additional hour of growth at 37°C was allowed to ensure metabolic consumption of unlabeled nutrients, after which the cultures were induced with IPTG and transferred to 20°C as described above. In the case of the isotopically labeled calmodulin, the N-terminal polyhistidine tag was proteolytically removed for use in NMR experiments by incubation with in-house produced Tobacco Etch Virus protease (TEV) for 20 hours at 4°C. Briefly, the protease itself also contains a histidine tag which allows selective removal of the protease and cleaved tag after a second pass through a Ni^2+^ column, and separation of tag-free calmodulin in the flow-through.

### Circular dichroism

Folding and stability of wild-type and hybrid cFLIP_L_ DED1 and DED2 constructs were assessed by circular dichroism (CD). All samples contained 3–6 μM protein in 1x PBS, pH = 7.5. CD scans were acquired at 20°C with four accumulations each in the 250–200 nm UV range, at 100 nm/min, and with a 1 nm bandwidth. CD melting curves were monitored at 222 nm between 20°C and 80°C, with a temperature ramp rate of 1°C/min (for DED2, the curve was monitored up to 90°C due to the higher melting point of this construct).

### Pull-down assays

50 μL aliquots of calmodulin-conjugated beads (Cat # 214303-52, Stratagene, La Jolla, CA) were equilibrated by washing the beads with reaction buffer (PBS, 2 mM CaCl_2_, 0.5% Tween-20, pH 7.4). The beads were suspended in 1 mL buffer and sedimented by centrifugation (< 6,000 rpm) three consecutive times. The reactions were assembled in a final volume of 0.5 mL, with normalized amounts of pure recombinant protein constructs (10 μg), and then incubated on a nutator at room temperature for two hours. After incubation the beads were sedimented at slow speeds (< 6,000 rpm) and washed successively three times. The supernatant and the pull-downs were analyzed by SDS-PAGE/Coomassie. Equal amounts of bait protein (calmodulin) in each reaction were ensured by using equal volumes of calmodulin-conjugated slurry. Conversely, equal amounts of prey were ensured by quantifying recombinant protein concentrations through A_280_ UV measurements. Recombinant protein purity was assessed through SDS-PAGE analysis. When appropriate, A_280_ results were followed up with image quantitation of SDS-PAGE bands using NIH’s ImageJ software.

The pull-downs with NCI-H2030 cell lysates were performed similarly. Briefly, 100 μg total lysate protein (as determined through a quantitative Bradford assay) was loaded per reaction. These cells did not express cFLIP_L_ to a level detectable in our Western Blot analyses. Therefore, the reactions were supplemented with 10 g cFLIP_L_. The results were analyzed by Western blotting.

### Western blots

Assays were performed according to standardized protocols [52]. The primary anti-cFLIP antibody (sc5276, Santa Cruz Biotech, Santa Cruz, CA) was diluted 1:1,000 in blocking solution (PBS, 0.1 mg/ml BSA fraction V) and the PVDF blot incubated for two hours at room temperature, followed by three successive washes in 10 mL TBST (TBS, 0.1% Tween-20), each wash lasting ten minutes. The secondary anti-mouse HRP-conjugated antibody (sc2031, Santa Cruz Biotech, Santa Cruz, CA) was diluted 1:10,000 in PBS, and the blot incubated for two hours at room temperature. Following this incubation, the blot was washed three times as previously. Chemiluminescence imaging was performed using a BioRad ChemiDoc XRS instrument, using default auto-exposure parameters.

### ELISA experiments

In all assays described, His-tagged calmodulin was immobilized on high-binding ELISA 96-well microplates (Greiner Bio-One, cat# 655061) by over-night incubation at 4°C of 90 nM protein in 50 mM sodium citrate buffer, pH = 5.0. The tested protein always carried a GST fusion tag, which enabled quantitation by fluorescence detection through the use of a rabbit IgG anti-GST AlexaFluor 488 conjugated antibody (Life Technologies, cat# A-11131).

The signal from the antibody fluorophore was detected using a Tecan Infinite F500 plate reader. After calmodulin immobilization, the plate wells were blocked with 1x PBST, 5% skim milk, 3% BSA, pH = 7.4. Secondary proteins were allowed incubation periods of two hours at room temperature, under gentle shaking (approximately 50 rpm). The antibody was allowed an incubation period of one hour at room temperature, under light-shielding conditions. Between each pair of steps, the wells were washed three times with 1x PBST (0.05% Tween-20), 50 μM calcium chloride, pH = 7.4. In the direct binding assays, the secondary protein was titrated by means of serial dilution in 100 mM Tris-HCl, 10 mM sodium phosphate, 10 mM sodium citrate, 150 mM sodium chloride, 0.5% Tween-20, 10% Superblock, 1% BSA, 100 μM calcium chloride, pH = 7.4. In the ELISA competition experiment, the concentration of GST-DED1 was held constant at the value of the derived K_D_ and the R4 peptide was titrated. All experiments were conducted in triplicate. K_D_ and IC_50_ values were obtained through data fitting performed in KaleidaGraph using a four-parameter logistic model, according to the sigmoidal dose-response fitting function:

$$\begin{array}{c} y=m_{1}+\frac{\left(m_{2}-m_{1}\right)}{1+{\left(\frac{x}{m_{3}}\right)}^{m_{4}}} \end{array}$$ (1)

where m_1_ and m_2_ are the fluorescence intensity values at zero and maximum analyte concentrations, respectively, x is the analyte concentration, m_3_ is the inflection point of the calibration curve, and m_4_ is the slope factor.

### NMR

All NMR experiments were carried out on a Bruker Avance 700 MHz spectrometer equipped with a TCI cryoprobe. For the backbone assignment of calmodulin, spectra were acquired at 30°C using a 290 μM sample of ^15^N,^13^C-calmodulin in 20 mM sodium phosphate, 150 mM sodium chloride, 1 mM calcium chloride, pH = 6.5. The backbone assignment was obtained from a set of triple resonance experiments comprising an HNCA, HN(CO)CA, HNCO, HN(CA)CO, HNCACB, CBCA(CO)NH, a 3D ^15^N-edited NOESY-HSQC, as well as a 2D ^15^N-HSQC spectrum [53]. Based on these experiments, the backbone assignment of calcium-bound human calmodulin was carried out using CARA and PACES [54, 55].

For the R4 peptide titration experiments, a 55 μM sample of ^15^N-labeled calmodulin in 24 mM sodium phosphate, 140 mM sodium chloride, 2.7 mM potassium chloride, 660 μM calcium chloride, pH = 7.0 was used. Experiments were carried out at 25°C. The calmodulin backbone assignment obtained for the doubly labeled sample described earlier was readily transferred to the control calmodulin spectrum acquired here (i.e., in the absence of R4). Subsequently, 118 residue assignments were unambiguously transferred to the spectrum acquired in the presence of a five-fold excess of R4 by gradually monitoring their shifts in response to increasing concentrations of R4 peptide (starting from 1:1 and up to 5:1 peptide to protein). The remaining signals experienced either too severe broadening or overlap in the presence of the peptide and could not be unambiguously assigned in the HSQC spectra of R4-bound calmodulin.

In order to ascertain which calmodulin residues are involved in binding to R4, Euclidean distances were calculated for the 118 assigned residues, as described by Williamson [56] as follows:

$$\begin{array}{c} d=\sqrt{\left(\frac{1}{2}\cdot \left[\delta_{H}^{2}+\left(\alpha \cdot \delta_{N}^{2}\right)\right]\right)} \end{array}$$ (2)

where δ_H_ and δ_N_ represent the chemical shifts of the ^1^H and ^15^N nuclei, respectively, and α is a scaling factor. An α value of 0.14 was used for all residues except glycine, for which α = 0.20 was employed [56]. The standard deviation σ of the Euclidean distance data set was calculated and residues for which the chemical shift change was greater than σ were identified, as well as residues for which the shift was greater than 2σ.

The R4 peptide resonances were assigned using TOCSY and ROESY spectra on a 0.5 mM peptide sample in 20 mM sodium phosphate, 150 mM sodium chloride, 1 mM calcium chloride, pH 6.5 [43, 44]. ^15^N-filtered transferred NOESY experiments were acquired on a sample containing 20 μM ^15^N-calmodulin and a 100:1 excess R4 peptide using a 250 ms mixing time [42]. All experiments were carried out at 15°C. Several peptide-to-protein ratios were assayed (starting with a 20:1 excess) until the resulting peptide resonances became narrow enough to allow deconvolution of the spin systems in the two-dimensional experiments. Spectra were acquired at several temperatures between 15 and 45°C, but the ones carried out at 15°C displayed the highest level of signal resolution (significant overlap at higher temperatures and the absence of the second residue spin system made resonance assignment more challenging).

### Structural characterization

The structure of the DED1 R4 peptide while bound to calmodulin was solved utilizing NOE distance constraints obtained from the ^15^N-filtered transferred NOESY. Structure calculation utilized the experimental distance constraints and a home-written distance geometry protocol which uses random metrization and refinement in four dimensions [57]. The distance between the two resolved Phe65 CH2 signals (1.78 Å) was used as an internal reference. Sequential and medium-range distance constraints were employed to calculate the solution structure of the R4 peptide. 97 structures were successfully calculated and 21 lowest-energy scoring structures were subsequently subjected to energy minimization in Chimera employing the AMBERff12SB force field and using 200 steps of steepest descent and 10 conjugate gradient steps, with a step size of 0.02 Å [58]. NOE restraint analysis and statistics for this ensemble were carried out using AQUA3.2 and PROCHECK-NMR [59]. A representative structure from the set was used to generate the R4-calmodulin complex model.

The resulting R4 structure was manually aligned in the binding pocket of calmodulin (PDB ID: 1QTX), in the binding region of the smooth muscle myosin light chain kinase peptide (sMLCK). The R4 peptide F^65^ and I^71^ residues were used as hydrophobic anchors in the appropriate calmodulin hydrophobic surface cavities. The alignment was refined relative to the sMLCK template by taking into account the chemical shift perturbation data from the NMR titration experiments. The starting structure was solvated and ionized in VMD [60]. A water box extending 8 Å beyond the protein/peptide complex was generated and 12 Na^+^ ions were added to neutralize the system. The protein and peptide backbone atoms, as well as the calmodulin Ca^2+^ ions were fixed, and the complex was subsequently optimized in NAMD [61]. Energy minimization was carried out at 298 K for 100 ps, followed by molecular dynamics for 100 ps using a CHARMM27 force field and a 2 fs step size.

### Homology modeling

The homology model for cFLIP DED1 was generated using the SWISS-MODEL workspace server by aligning cFLIP residues 4–73 with the MC159 viral FLIP template (PDB ID: 2BBR.1.A). The query and template had 36.76% sequence identity. The resulting model had a QMEAN score of -1.86 and a GMQE score of 0.69.

### BLAST screen

All 180 peptide sequences deposited in the Calmodulin Target Database were screened against the amino acid sequence of cFLIP DED1. The search parameter settings were as follows: word size 2, filters&masking *Off*, compositional adjustments *Off*, score matrix PAM30, gap costs: *existence* 7, and *extension* 2. The clustering was analyzed with Clustal W [62].

## Supporting Information

S1 Fig Schematic depiction of the cFLIP and calmodulin constructs used in the study. Domain boundaries, fusion protein partners, and protease cleavage sites are marked for each protein construct employed. “cFLIP_FL_” is the *full-length* cFLIP long isoform.
(TIF)

S2 Fig Identification of the calmodulin binding site on cFLIP DED1. **(A)** BLAST analysis of the Calmodulin Target Database revealed hits on DED1, which clustered on regions 2 and 4. Mostly positive and hydrophobic amino acids are conserved across the series. **(B)** Helical projections of the peptides corresponding to DED1 regions 2 and 4 reveal an amphipathic structure with positive charges, typical of most known calmodulin binding peptides. By contrast, the sequence of region 3 has a scrambled projection, which correlates with no binding activity (see Fig 3C).
(TIF)

S3 Fig Circular dichroism spectra of DED1 and DED2 constructs. All DED1 and DED2 wild-type and hybrid constructs were analyzed by circular dichroism (CD) to ensure proper protein folding. CD scans demonstrate the expected helical character for all constructs. Melting curves (monitored at 222 nm) indicate the hybrid DED1-DED2 swap chimeras maintain folding and thermodynamic stability.
(TIF)

S4 Fig The cFLIP/calmodulin interaction can be inhibited in cell lysates. In H2030 lung cancer lysates binding of cFLIP to calmodulin is strongly inhibited by **(A)** 2 mM EDTA, and weakly inhibited by **(B)** 1 mM R4 peptide.
(TIF)

S1 Table Table of primers. Primer sequences used to generate all constructs used in this article are listed, as well as the plasmids in which the constructs were introduced.
(PDF)

S2 Table NMR restraint analysis for R4 peptide. The structure of the DED1-derived calmodulin-binding peptide, R4, was calculated from homonuclear ^1^H-^1^H NOE restraints alone, using an in-house distance geometry protocol coupled with simulated annealing and energy minimization. This table summarizes the analysis of these distance restraints, carried out using AQUA3.2 and PROCHECK-NMR.
(PDF)
