# Supplementary figures and images for: Identification and Characterization of the Interaction Site between cFLIPL and Calmodulin
Source: PLoS One. 2015 Nov 3;10(11):e0141692. doi: 10.1371/journal.pone.0141692 (PMC4631386; doi:10.1371/journal.pone.0141692)

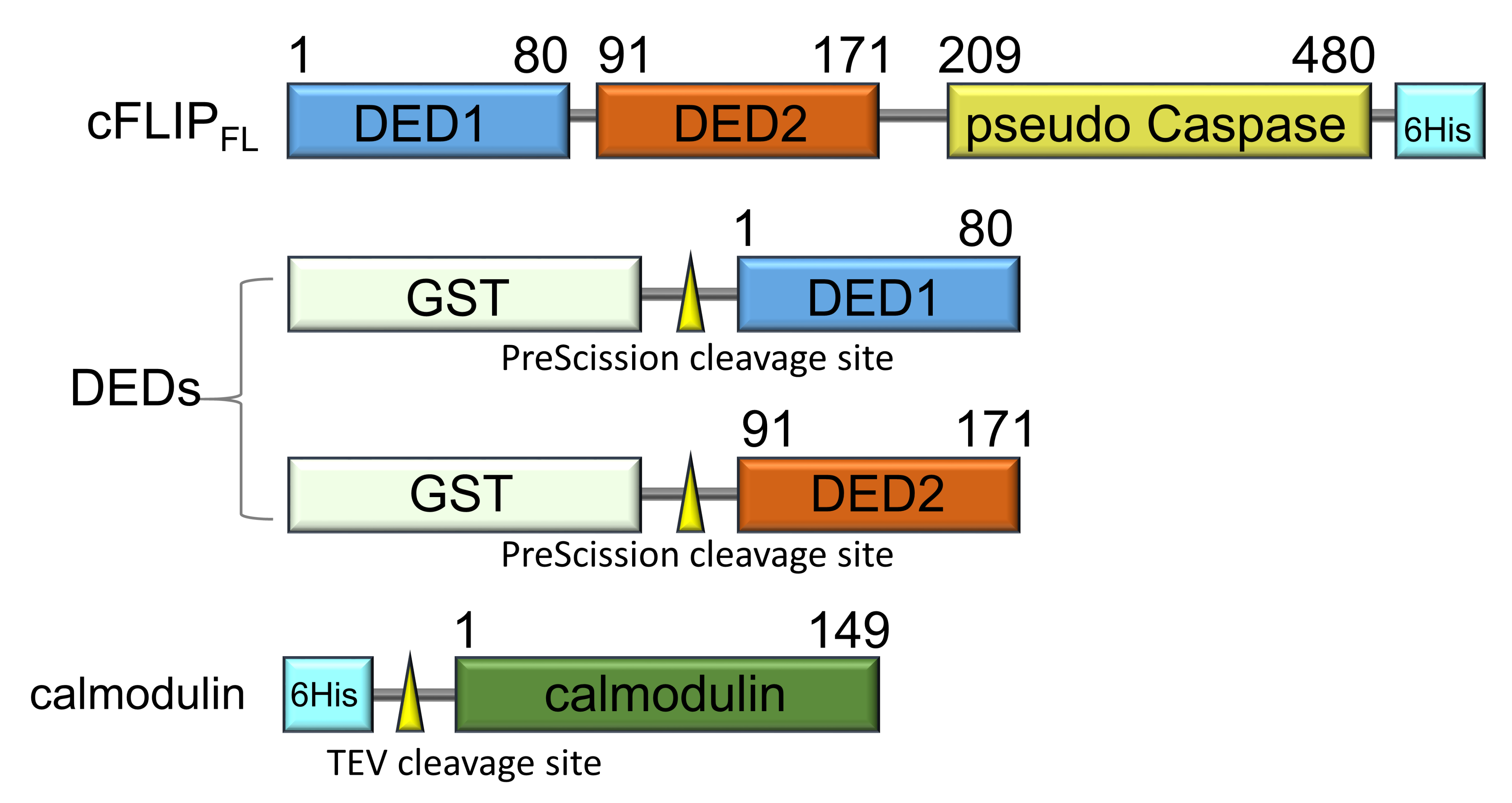

Supplement: S1 Fig — Domain boundaries, fusion protein partners, and protease cleavage sites are marked for each protein construct employed. “cFLIPFL” is the full-length cFLIP long isoform. (TIF) [file pone.0141692.s001.tif]

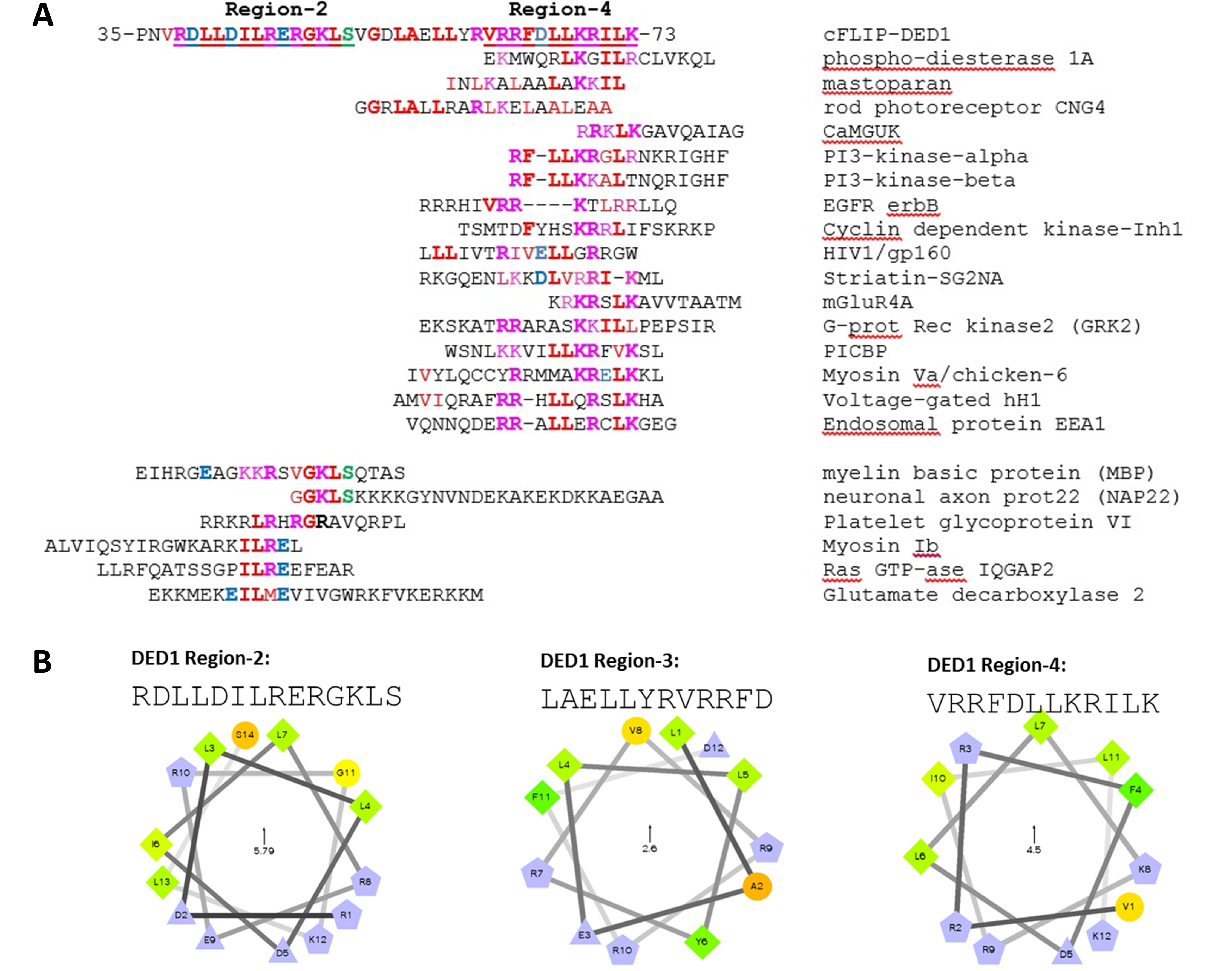

Supplement: S2 Fig — (A) BLAST analysis of the Calmodulin Target Database revealed hits on DED1, which clustered on regions 2 and 4. Mostly positive and hydrophobic amino acids are conserved across the series. (B) Helical projections of the peptides corresponding to DED1 regions 2 and 4 reveal an amphipathic structure with positive charges, typical of most known calmodulin binding peptides. By contrast, the sequence of region 3 has a scrambled projection, which correlates with no binding activity (see Fig 3C). (TIF) [file pone.0141692.s002.tif]

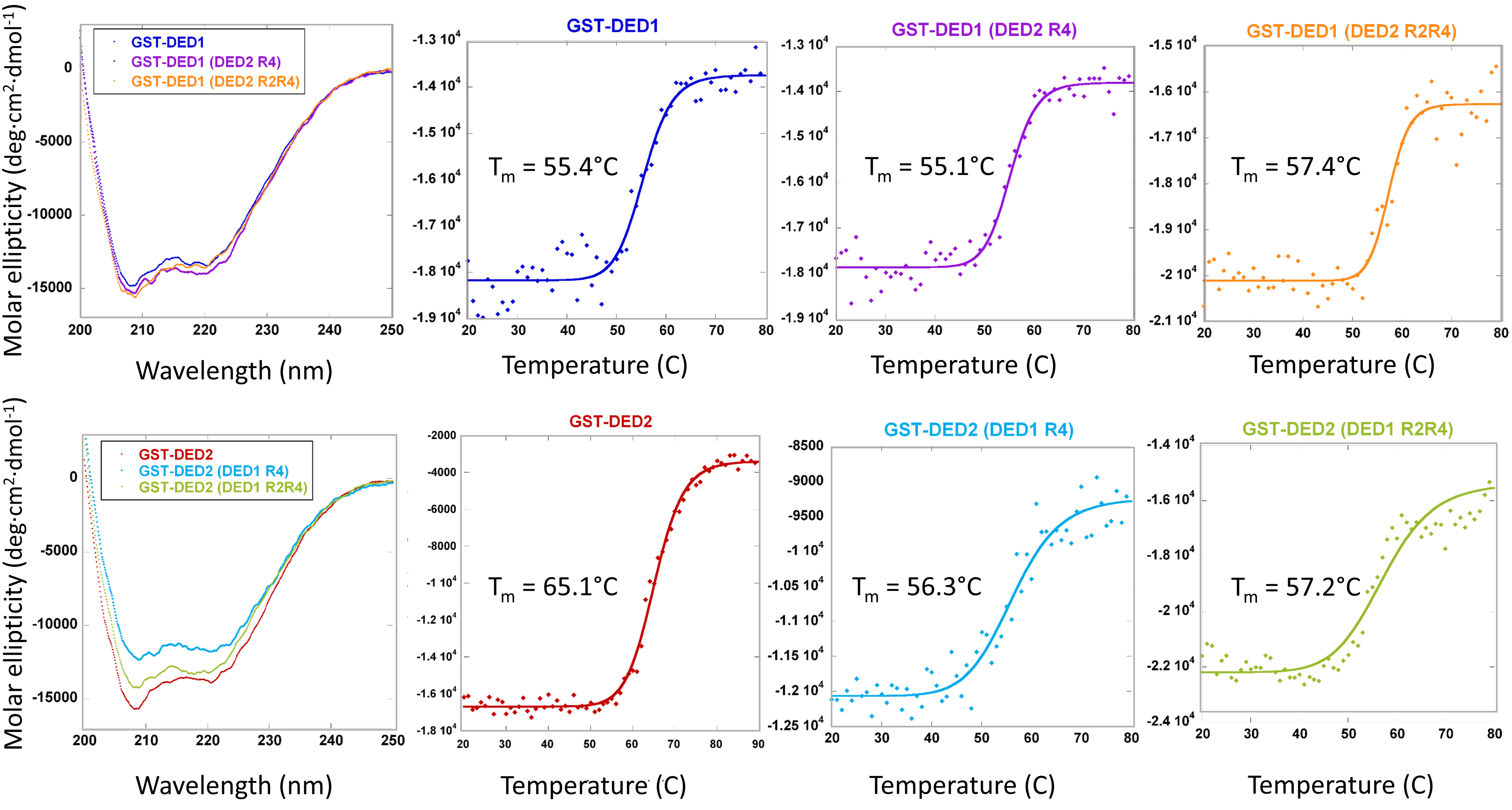

Supplement: S3 Fig — All DED1 and DED2 wild-type and hybrid constructs were analyzed by circular dichroism (CD) to ensure proper protein folding. CD scans demonstrate the expected helical character for all constructs. Melting curves (monitored at 222 nm) indicate the hybrid DED1-DED2 swap chimeras maintain folding and thermodynamic stability. (TIF) [file pone.0141692.s003.tif]

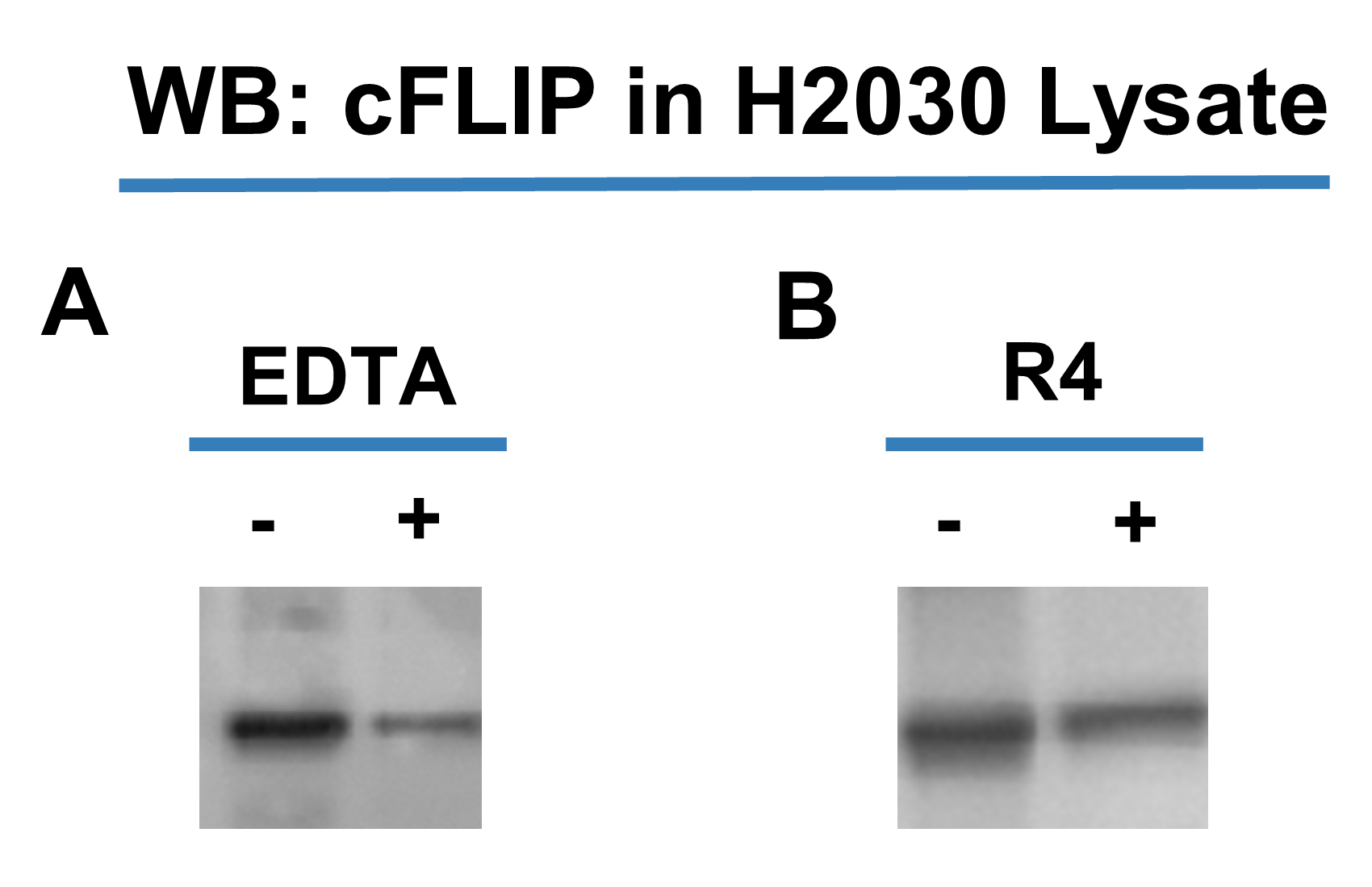

Supplement: S4 Fig — In H2030 lung cancer lysates binding of cFLIP to calmodulin is strongly inhibited by (A) 2 mM EDTA, and weakly inhibited by (B) 1 mM R4 peptide. (TIF) [file pone.0141692.s004.tif]
