# Supplementary material for: Identification and Characterization of the Interaction Site between cFLIPL and Calmodulin
Source: PLoS One. 2015 Nov 3;10(11):e0141692. doi: 10.1371/journal.pone.0141692 (PMC4631386; doi:10.1371/journal.pone.0141692)
Supplement: S1 Table — Primer sequences used to generate all constructs used in this article are listed, as well as the plasmids in which the constructs were introduced. (PDF) [file pone.0141692.s005.pdf]

Construct	Plasmid	Primers
cFLIP-6His	pET21 b+ ΔT7 (modified)*	F: 5'-atacatatgtctgctgaagtcatccatcaggttgaa-3'<br>R: 5'-atacatatgtgtgtaggagaggataagtttctttctcagagt- 3'
GST-cFLIP-DED1	pGEX-6P-1	F: 5'-ctggaagttctgttccaggggcccatgtctgctgaagtcatccatcaggtt-3'<br>R: 5'-cgcgaggcagatcgtcagtcagtcactccacagcttttctgtccatcttcaa-3'
GST-cFLIP-DED2	pGEX-6P-1	F: 5'-ctggaagttctgttccaggggccctcggactatagagtgctgatggca-3'<br>R: 5'-cgcgaggcagatcgtcagtcagtcagtacttctggatttttgtcttcaggtc-3'
GST-cFLIP-DED1 (DED2-R4 swap)	pGEX-6P-1	F: 5'-gtcggggacttggctgaactgctctacagaatccacagaatagacctgaagacaaaaatccagaag-3'<br>R: 5'-cgcgaggcagatcgtcagtcagtca-3'
GST-cFLIP-DED1 (DED2-R2R4 swap)	pGEX-6P-1	F: 5'-cgggatgttgctatagatgtggttccacctaatgtcaagagtttcttggaccttgtggttgagttggagaaacta-3'<br>R: 5'-cgcgaggcagatcgtcagtcagtca-3'
GST-cFLIP-DED2 (DED1-R4 swap)	pGEX-6P-1	F: 5'-gccccagatcaactggatttattagaaaaatgcctaaagaacgtgaggcgatttgacctgctcaaacgtatcttgaag-3'<br>R: 5'-cgcgaggcagatcgtcagtcagtca-3'
GST-cFLIP-DED2 (DED1-R2R4 swap)	pGEX-6P-1	F: 5'-tacatgggccgaggcaagataagcaaggagagggaccttctggatattttacgggaaagaggt-3'<br>R: 5'- cgcgaggcagatcgtcagtcagtca - 3'
6His-calmodulin	pET-16b (modified)*	F: 5'-agcggcgagaacctgtactttcagggaatggctgaccaactgactgaagagcagat-3'<br>R: 5'-gggctttgttagcagccggatctcactttgctgtcatcatttgtacaaactcttcatagt-3'

"F" and "R" denote forward and reverse primers, respectively.
* See 'Materials and Methods' for details on engineered modifications.
