# Supplementary material for: Identification and Characterization of the Interaction Site between cFLIPL and Calmodulin
Source: PLoS One. 2015 Nov 3;10(11):e0141692. doi: 10.1371/journal.pone.0141692 (PMC4631386; doi:10.1371/journal.pone.0141692)
Supplement: S2 Table — The structure of the DED1-derived calmodulin-binding peptide, R4, was calculated from homonuclear 1H-1H NOE restraints alone, using an in-house distance geometry protocol coupled with simulated annealing and energy minimization. This table summarizes the analysis of these distance restraints, carried out using AQUA3.2 and PROCHECK-NMR. (PDF) [file pone.0141692.s006.pdf]

**NOE restraints**	80
Intra-residue	36
Sequential	23
Medium range ($\|i - j\| \leq 4$)	21
Long range ($\|i - j\| > 4$)	0
RMSD from experiment (Å)	1.16 ± 0.32
**Structure statistics**	21 structures
**NOE violations**	
Average overall violation (Å)	0.32 ± 0.53
Average intra-residue violation (Å)	0.14 ± 0.33
Average sequential violation (Å)	0.40 ± 0.50
Average medium range violation (Å)	0.54 ± 0.69
**Ramachandran analysis**	
Most favored regions (%)	58.10
Additional allowed regions (%)	32.90
Generously allowed regions (%)	8.60
Disallowed regions (%)	0.50
**Average pairwise RMSD***	
Overall (Å)	1.41
Secondary structure (residues 5-10) (Å)	0.83

Statistics obtained from AQUA3.2 and PROCHECK-NMR analyses.
* RMSDs computed in UCSF Chimera.
